# Financial hardship and mental health among cancer survivors during the COVID-19 pandemic: An analysis of the US COVID-19 Household Impact Survey

**DOI:** 10.3389/fpubh.2022.946721

**Published:** 2022-11-22

**Authors:** Jessica Y. Islam, Kea Turner, Huda Saeb, Margaux Powell, Lorraine T. Dean, Marlene Camacho-Rivera

**Affiliations:** ^1^Cancer Epidemiology Program, H. Lee Moffitt Cancer Center and Research Institute, Tampa, FL, United States; ^2^Department of Oncological Sciences, University of South Florida, Tampa, FL, United States; ^3^Health Outcomes and Behavior Department, H. Lee Moffitt Cancer Center and Research Institute, Tampa, FL, United States; ^4^Department of Gastrointestinal Oncology, H. Lee Moffitt Cancer Center and Research Institute, Tampa, FL, United States; ^5^Morehouse School of Medicine, Atlanta, GA, United States; ^6^Department of Epidemiology, Bloomberg School of Public Health, Johns Hopkins University, Baltimore, MD, United States; ^7^Department of Oncology, School of Medicine, Johns Hopkins University, Baltimore, MD, United States; ^8^Department of Community Health Sciences, SUNY Downstate Health Sciences University, Brooklyn, NY, United States

**Keywords:** cancer survivors, COVID-19, SARS-CoV-2, pandemic, financial hardship, mental health, depression, anxiety

## Abstract

**Purpose:**

Our objective was to (1) identify associated characteristics of financial hardship (FH), and (2) evaluate associations of FH with mental health symptoms among cancer survivors during the COVID-19 pandemic.

**Methods:**

Using data from the nationally representative COVID-19 Impact Survey, we defined cancer survivors as those with a self-reported diagnosis of cancer (*n* = 854,7.6%). We defined FH using the following question: “Based on your current financial situation, how would you pay for an unexpected $400 expense?” Multivariable Poisson regression was used to estimate adjusted prevalence ratios (aPR) with 95% confidence intervals (95%CI) to identify associated characteristics of FH and associations of FH with mental health symptoms among cancer survivors overall and by age (18–59 years/60+ years).

**Results:**

Forty-one percent of cancer survivors reported FH, with 58% in 18–59 and 33% in 60+ year old respondents. Compared to cancer survivors aged 60+ years, those aged 30–44 (aPR:1.74,95% CI:1.35–2.24), and 45–59 years (aPR:1.60,95% CI:1.27–1.99) were more likely to report FH. Compared to non–Hispanic(NH)–White cancer survivors, NH–Black cancer survivors had a 56% higher prevalence of FH (aPR:1.56; 95% CI: 1.23–1.97). Among 60+ years aged cancer survivors, NH–Black (aPR:1.80; 95% CI: 1.32–2.45) and NH–Asian cancer survivors (aPR:10.70,95% CI:5.6–20.7) were more likely to experience FH compared to their NH–White counterparts. FH was associated with feeling anxious (aPR:1.51,95% CI:1.11–2.05), depressed (aPR:1.66,95% CI:1.25–2.22), and hopeless (aPR:1.84,95% CI:1.38–2.44).

**Conclusion:**

Minoritized communities, younger adults, and cancer survivors with low socioeconomic status had a higher burden of FH, which was associated with feelings of anxiety, depression, and hopelessness.

## Introduction

In the United States, as of November 2022, the COVID-19 pandemic has resulted in over one million deaths and over 97 million cases of SARS-CoV-2, the infection that leads to COVID-19, since early 2020 (1). The pandemic led to significant changes in everyday life, including behavioral changes to curb the spread of SARS-CoV-2, such as social distancing, wearing of face masks, and quarantining (2–4), particularly among adults with chronic conditions, such as cancer survivors, due to their increased risk of COVID-19 associated morbidity. In addition to these social changes, the US has experienced a significant economic impact, including the highest unemployment rates since World War II (5, 6). Almost half of US adults have reported either they or someone in their household has experienced a loss of employment or experienced a reduction in salary due to the pandemic (7). Cancer patients may be particularly vulnerable to financial hardship during the pandemic from both costs of cancer care and financial strains imposed by the pandemic. In fact, a recent study found that 50% of gynecological cancer patients treated at a hospital in New York City (NY) reported feeling more financial stress since the start of the pandemic (8).

Financial strain or hardship among cancer survivors is of particular concern given the high costs associated with cancer treatment, including survivorship care. Engagement in high quality cancer survivorship care is vital to extending survival, improving quality of life, and surveillance for recurrence detection or progression of diagnosed cancers. One of the major barriers to engagement in survivorship care is cost, specifically lack of reimbursement structures/insurance coverage for survivorship care services (9). Prior research focused on cancer costs associated with active treatment in the US demonstrate that cancer care spending is projected to grow from $183 billion in 2015 to $246 billion by 2030, an increase by over one-third (10). The average monthly out-of-pocket spending of patients undergoing active cancer treatment range from $180 to $2,600 per month in the US (11), and costs continue beyond the active treatment phase (12). In addition to high out-of-pocket spending, cancer survivors are at risk for productivity losses due to employment disruption (13). As a consequence, cancer survivors are at risk for medical debt, bankruptcy, and financial distress (14, 15), with long-term impacts on consumer credit (16). A systematic review illustrated that approximately half of US cancer patients reported experiencing financial distress and psychologic stress during the pre-pandemic period, with 47–49% of survivors reporting some form of financial distress (17). Recent work based on the 2013–2018 National Health Interview Survey (NHIS) demonstrates that cancer survivors frequently report financial worry, which was also associated with psychological distress (18). Financial hardship among cancer survivors may have been exacerbated during the COVID-19 pandemic. The COVID-19 pandemic led to increased healthcare costs [e.g., COVID-related care costs (19)] and additional financial strain (e.g., pandemic-related unemployment, productivity losses, and health insurance coverage disruption) for many US adults (20). Given the extent to which the pandemic affected the US economy, research is needed to better understand how COVID-19 has affected financial hardship among cancer survivors in the US. Research during the pandemic period suggests that cancer survivors may have been disproportionately affected by rising unemployment (20) potentially leading to loss of insurance and income. Additionally, the economic impact of the pandemic on vulnerable populations of cancer survivors, specifically adolescent or young adult cancer survivors (21, 22) and those with low-income (8), has been evaluated in the pandemic period. However, to our knowledge, limited prior research (20) has been conducted nationally among a representative sample of cancer survivors to evaluate financial hardship and the potential impacts on mental health during the pandemic, particularly among older cancer survivors.

To address this gap, this study will evaluate (1) the prevalence and associated characteristics of financial hardship and (2) the association between financial hardship and self-reported mental health symptoms among younger (< 60 years) and older (≥60 years) US cancer survivors during the COVID-19 pandemic. Information from this study may inform future policies and interventions to address financial hardships and poor mental health among cancer survivors in the context of the ongoing public health crisis in the US.

## Methods

### COVID-19 impact survey

Data for these analyses were obtained from the publicly available COVID-19 Household Impact Survey, conducted by the nonpartisan and objective research organization NORC at the University of Chicago (23). The COVID-19 Household Impact Survey provides national and regional statistics about physical health, mental health, economic security, and social dynamics in the US (24). The pooled cross-sectional survey is designed to provide estimates of the US adult (ages 18 and older) household population nationwide and for 18 regional areas including 10 states (CA, CO, FL, LA, MN, MO, MT, NY, OR TX) and 8 Metropolitan Statistical Areas (Atlanta, Baltimore, Birmingham, Chicago, Cleveland, Columbus, Phoenix, Pittsburgh). For these analyses, we pooled cross-sectional national data collected during Week 1 (April 20-26, 2020), Week 2 (May 4-10, 2020), and Week 3 (May 30th–June 8th, 2020), based on data availability. Details regarding the dataset and data collection methods have been previously published (25, 26).

### Study population

The COVID-19 Impact Survey was administered through the AmeriSpeak^®^ panel, which is designed to be representative of the US population. The sampling frame covers ~97% of US households. The sampling strategy includes random selection of US households using area probability and address-based sampling from the NORC National Sample Frame. The sampled households were contacted by US mail, and telephone to allow for multiple modalities for survey participation (e.g., if a participant does not have internet). The surveys are conducted in English and Spanish. In households with more than one adult, only one was selected at random for the sample. The average survey response rate across weeks 1–3 was 21.8%.

### Cancer survivors

We defined cancer survivors as those participants with a self-reported cancer diagnosis. Participants were asked the following question: “Has a doctor or other health care provider ever told you that you have any of the following: Diabetes; High blood pressure or hypertension; Heart disease, heart attack or stroke; Asthma; Chronic lung disease or COPD; Bronchitis or emphysema; Allergies; a Mental health condition; Cystic fibrosis; Liver disease or end-stage liver disease; Cancer; a Compromised immune system; or Overweight or obesity.” We defined those who selected “Cancer” as a cancer survivor, similar to our previously published work (26).

### Primary measures

Our primary measures for this analysis were financial hardship and mental health symptoms. We defined financial hardship using the following question: “Suppose you have an unexpected expense that costs $400. Based on your current financial situation, how would you pay for this expense?” The following options were provided to respondents and they were able to select all that apply: (1) put it on my credit card and pay it off in full at the next statement; (2) put in on my credit card and pay it off over time; (3) use money currently in my checking or savings account or with cash; (4) use money from a bank loan or line of credit; (5) borrow from a friend or family member; (6) use a payday loan, deposit advance or overdraft; (7) sell something; and (8) I would not be able to pay for it right now. Respondents were categorized as experiencing financial hardship if they only chose any of the following options: put it on my credit card and pay if off over time; use money from a bank loan or line of credit; I wouldn't be able to pay for it right now; sell something; use a payday loan, deposit advance or overdraft; borrow from a friend or family member. We used this definition based on prior research conducted by the U.S. Federal Reserve in the general population (27).

Next, to evaluate mental health symptoms, participants were asked: “In the past 7 days, how often have you? (1) Felt nervous anxious or on edge, (2) Felt depressed, (3) Felt lonely, and (4) Felt hopeless about the future.” Participants were able to choose from the following list of options for each mental health symptom: (1) Not at all or less than 1 day; (2) 1–2 days, (3) 3–4 days, and (4) 5–7 days. For multivariable analyses stratified by age group, we recategorized self-reported mental health symptoms to either not at all or < 1 day or 1–7 days per week due to sample size concerns and to avoid small cell sizes to effectively conduct regression modeling.

### Covariates

Prior studies suggest that factors such as household income and education level are associated with financial hardship among cancer survivors (28–31). Therefore, we selected covariates that have demonstrated a prior relationship with financial hardship, including: age (18–59, 60+), gender (male/female), marital status (married/living with a partner, widowed/divorced/separated, never married), race/ethnicity categories [non-Hispanic (NH) White, NH- Black, Hispanic, NH-Asian, NH-Other], education categories (no high school diploma, HS graduate or equivalent, some college, baccalaureate degree or above), household income (< $30,000;$30,000- < $50,000;$50,000- < $75,000; $75,000- < $100,000;≥$100,000), population density (rural, suburban, urban), census region (Northeast, Midwest, South, West), any comorbid chronic conditions, (yes/no), and insurance type (purchased plan/employer-sponsored /TRICARE/Medicaid/Medicare/Dually-eligible/VA/uninsured). Detailed information regarding employment status was available to delineate the following employment categories: employed in the last 7 days, retired, or not interested in working at this time, or under/unemployed due to COVID-19 or unable to find employment.

### Data analyses

Descriptive statistics were summarized, by cancer survivorship status and age categories, in percentages among all respondents with 95% confidence intervals (CIs). We present the study results stratified by age group as prior research conducted in the pre-pandemic period has demonstrated that the prevalence and associated characteristics of financial hardship experienced by cancer survivors vary by age category due to social and behavioral factors (e.g., insurance status, employment status) (17, 32). To identify demographic groups that may be more likely to report financial hardship, we estimated associated characteristics of financial hardship among cancer survivors. We computed prevalence ratios with Poisson regression using robust estimation of standard errors (33–35). Potential variables for inclusion in the model were assessed using available sociodemographic variables and unadjusted Poisson regression analysis. Due to the exploratory nature of this analysis using a predictive framework, a *p* < 0.10 was used as criteria for variable selection in the multivariable Poisson regression model. For multivariable Poisson regression models, adjusted prevalence ratios (aPR), and 95% CIs for each independent variable were calculated.

Next, we used multivariable Poisson regression to assess associations between financial hardship and self-reported mental health symptoms experienced at least 1 day in the last week. We adjusted for survey week, age (when appropriate excluding age-stratified models), sex, race/ethnicity, annual household income, education, insurance status, employment status, and area of residence (urban/rural). To address concerns regarding existing mental health symptoms before the COVID-19 pandemic, we conducted a sensitivity analysis to evaluate mental health symptoms among those without a history of a mental health condition based on self-report. We were able to assess the history of a mental health condition through the following question: “Has a doctor or healthcare provider ever told you that you have any of the following?”, which includes a response option for mental health condition. Based on the exploratory nature of this analysis, we did not include an adjustment for multiple comparisons (36, 37). Missing data were minimal (< 3% of observations), and we used a complete case approach. All statistical analyses were conducted using Stata IC 15 (StataCorp LLC, College Station, TX). Sampling weights were applied to provide results that were nationally representative of the U.S. adult population. We conducted a sensitivity analysis and repeated our analyses using fixed-effects multivariable logistic regression modeling and have included those results in the Appendix. The analytic sample includes 10,760 adults nationwide.

## Results

Table 1 summarizes characteristics of the overall sample and our study population of interest, cancer survivors, stratified by age categories 18–59 years and 60+years. We provide sample characteristics of all adults and cancer survivors overall in Supplementary Table 1. Sixty percent of cancer survivors were over the age of 60 years. Sixty percent of cancer survivors aged 18–59 years were female, whereas 48% of those aged 60+ years were female. Cancer survivors aged 18–59 years and 60+ years were most frequently married or living with a partner (54, 59%), NH-White (70, 76%), at least some college education (64, 63%), and resided in urban areas (71, 64%). One-quarter of cancer survivors 18–59 years were unemployed due to COVID-19 or unable to find employment, and 72% of 60+ cancer survivors were retired or not interested in working at this time. Over one-third of 18–59 cancer survivors had a household income $100,000 or greater. Seventy percent of 60+ cancer survivors had a comorbid cardiometabolic condition. Twenty-one percent of 18–59 cancer survivors had an existing diagnosed mental health condition as compared to 9% among 60+ cancer survivors. Most 18–59 cancer survivors had either employer sponsored health insurance (62%) or Medicaid (33%). Seventy-seven percent of 60+ cancer survivors were Medicare insured. Twenty-one percent of 18–59 cancer survivors reported they would not be able to cover a $400 unexpected expense based on their current financial situation, compared to 10% among 60+ cancer survivors. About half of both 18–59 (49%) and 60+ (54%) cancer survivors reported they would be able to use money currently in their checking or savings account.

**Table 1:** Characteristics of COVID Impact Survey respondents (*n* = 10,760), a nationally representative survey of the US, stratified by cancer diagnosis (April-June 2020).

	**Total (*n =* 10,760)**				**Cancer survivors (*n =* 854)** ^*^			
	**18–59 years**		**60**+ **years**		**18–59 years**		**60**+ **years**	
	**Col %**	**95% CI**	**Col %**	**95% CI**	**Col %**	**95% CI**	**Col %**	**95% CI**
**Sex**								
Male	48.5	46.9, 50.2	48.0	45.7,50.2	39.9	32.7,47.6	51.7	46.4,57.0
Female	51.5	49.8, 53.1	52.0	49.8,54.3	60.1	52.4,67.3	48.3	43.0,53.6
**Marital status**								
Married/living with partner	57.7	56.1, 59.4	56.2	54.0,58.5	53.5	45.6,61.3	58.8	53.6,63.9
Widowed/divorced/separated	11.7	10.7, 12.6	34.5	32.3,36.6	27.6	21.1,35.4	33.1	28.5,38.2
Never married	30.6	29.0, 32.3	9.3	8.1,10.7	18.8	13.0,26.5	8.0	5.6,11.4
**Race/ethnicity**								
White, NH	57.9	56.2, 59.5	72.0	69.8,74.1	70.4	62.7,77.1	76.4	71.2,80.9
Black, NH	11.6	10.6, 12.6	11.6	10.2,13.3	10.9	6.6,17.6	12.0	8.4,16.9
Hispanic	6.3	5.4, 7.3	1.7	1.2,2.4	11.6	7.3,18.0	5.6	3.6,8.8
Asian, NH	19.3	17.9, 20.8	9.5	8.1,11.1	3.0	1.5, 6.1	0.6	0.1, 4.1
Other, NH	3.6	3.1, 4.1	3.5	2.8,4.4	1.5	0.7, 3.3	3.2	2.2, 4.7
**Employment status**								
Employed in the last 7 days	61.3	59.7, 62.9	22.0	20.3,23.9	57.7	49.8,65.3	17.2	13.8,21.2
Retired/not interested in working at this time	12.2	11.2, 13.3	68.6	66.5,70.6	17.3	12.4,23.7	71.9	66.9,76.5
Unemployed due to covid-19 or unable to find employment^†^	26.5	25.0, 28.0	9.3	8.1,10.7	24.9	18.3,33.0	10.9	7.7,15.2
**Education**								
No HS diploma	10.8	9.5, 12.1	7.0	5.7,8.6	8.3	4.6,14.4	5.4	3.2,8.7
Hs graduate	27.6	26.0, 29.2	29.5	27.4,31.7	27.4	20.3,35.9	31.9	26.8,37.4
Some college	27.5	26.2, 28.7	28.4	26.7,30.1	26.2	20.8,32.5	28.8	24.9,33.1
Baccalaureate or above	34.2	32.8, 35.7	35.2	33.0,37.4	38.1	30.9,45.8	33.9	29.1,39.1
**Household income**								
< $30,000	26.5	25.0, 28.1	27.4	25.4,29.5	23.5	17.1,31.3	28.8	24.0,34.1
$30,000- < $50,000	17.1	16.0, 18.3	22.5	20.7,24.4	18.5	13.4,25.0	23.4	19.2,28.1
$50,000- < $75,000	19.2	18.0, 20.5	17.1	15.6,18.8	13.3	9.4,18.5	19.0	15.3,23.5
$75,000- < $100,000	13.7	12.7, 14.8	13.1	11.7,14.8	8.1	5.1,12.4	10.9	8.2,14.4
≥$100,000	23.5	22.1, 24.9	19.8	18.1,21.6	36.7	29.4,44.7	17.9	14.5,21.9
**Region**								
Northeast	17.2	15.9, 18.5	17.9	16.1,19.8	18.9	12.8,26.9	16.3	12.7,20.6
Midwest	20.5	19.3, 21.7	21.5	19.9,23.3	23.7	17.9,30.7	21.7	17.9,26.2
South	37.8	36.2, 39.4	38.0	35.8,40.3	27.5	21.4,34.6	38.9	33.7,44.3
West	24.6	23.2, 26.0	22.5	20.8,24.3	29.9	23.3,37.5	23.1	19.2,27.5
**Population density**								
Rural	8	7.2, 8.9	11.5	10.1,13.1	7.3	4.7,11.2	16.7	12.5,21.9
Suburban	17.6	16.4, 18.8	21.3	19.6,23.1	21.7	16.2,28.4	19.1	15.4,23.3
Urban	74.4	73.0, 75.8	67.2	65.1,69.2	70.9	63.9,77.1	64.3	58.9,69.3
**Comorbid conditions**								
Cardiometabolic diseases^‡^	26.2	24.8, 27.6	65.0	62.9,67.1	41.8	34.3,49.6	70.3	65.4,74.8
Respiratory diseases^§^	23.3	21.9, 24.7	24.0	22.2,26.0	36.9	29.6,44.8	25.2	21.0,29.9
Overweight/obesity	32.3	30.8, 33.8	35.7	33.6,37.8	47.1	39.4,54.9	38.6	33.5,43.8
Mental health conditions	18.7	17.5, 20.0	7.5	6.4,8.7	21.7	15.8,29.2	8.7	6.1,12.2
**Insurance type or health coverage plans**								
Purchased plan	12.5	11.4, 13.7	28.3	26.3,30.5	10.4	6.6,16.0	25.6	21.3,30.3
Employer-sponsored	58.8	57.2, 60.5	35.4	33.3,37.6	62.1	54.1,69.5	38.5	33.5,43.8
Tricare	4	3.5, 4.7	6.8	5.8,7.9	1.9	1.0,3.8	9.5	6.6,13.5
Medicaid	22	20.7, 23.4	26.9	24.8,29.0	32.5	25.2,40.7	29.0	24.2,34.4
Medicare	5.7	5.0, 6.5	71.7	69.7,73.6	18.7	13.5,25.4	76.5	71.7,80.7
Dually eligible (medicare & medicaid)	4.5	4.0, 5.2	22.3	20.4,24.3	15.4	10.6,21.9	26.0	21.3,31.4
VA	3	2.5, 3.5	8.0	6.9,9.2	2.7	1.3,5.4	12.3	9.1,16.4
Indian health service	1.6	1.2, 2.2	0.2	0.1,0.5	0.7	0.2,2.2	0.1	0.0,0.3
No insurance	11.4	10.3, 12.5	2.6	2.0,3.3	3.8	2.0,7.1	2.5	1.1,5.4
**Financial hardship measure**								
**Suppose that you have an unexpected expense that costs $400. Based on your current financial situation, how would you pay for this expense? If you would use more than one method to cover this expense, please select all that apply**								
Put it on my credit card and pay it off in full at the next statement	29.1	27.7, 30.6	46.1	43.9,48.3	19.3	14.0,25.9	50.4	45.1,55.7
Put it on my credit card and pay it off over time	19	17.8, 20.3	17.6	16.0,19.4	20.4	14.9,27.3	16.3	13.1,20.2
Use money currently in my checking or savings account or with cash	48.5	46.9, 50.2	57.0	54.7,59.2	48.8	41.1,56.5	53.7	48.4,59.0
Use money from a bank loan or line of credit	2.9	2.4, 3.4	3.2	2.6,4.1	4.3	2.2,8.2	5.6	3.5,9.0
Borrow from a friend or family member	11.4	10.3, 12.5	4.4	3.5,5.6	14.1	9.0,21.5	2.7	1.7,4.2
Use a payday loan, deposit advance or overdraft	2.2	1.8, 2.8	1.1	0.7,1.5	1.9	0.8,4.7	0.4	0.2,1.2
Sell something	8.4	7.5, 9.4	3.8	3.0,4.8	6.3	3.7,10.4	3.3	1.9,5.7
I would not be able to pay for it right now	17.7	16.4, 19.1	9.9	8.6,11.5	20.6	14.6,28.4	10.2	6.7,15.1

* 2.46% of participants either chose: not sure, skipped or refused, when asked about their chronic conditions including cancer.

† Response Options: I was laid-off temporarily or furloughed, I was not at my usual jobs because I was caring for children not in school, I was not at my usual jobs because I was caring for an elderly person, I was not at my usual jobs because I was caring for someone with COVID-19, I was not at my usual jobs because I was recovering from COVID-19 or isolating due to exposure to COVID-19, I was unemployed but looking for work since March 1st, 2020 when COVID-19 began spreading in the US, I was unemployed and began looking for work after March 1, 2020 when COVID-19 began spreading in the US.

‡ Cardiometabolic conditions: diabetes, high blood pressure, heart disease, liver disease or end stage liver disease.

§ Respiratory conditions: Asthma, chronic lung disease or COPD, bronchitis, or emphysema.

### Prevalence and associated characteristics of financial hardship among cancer survivors

Overall, forty-two percent of cancer survivors reported financial hardship. By age group, 58% of 18–59 years and 33% of 60+ cancer survivors reported financial hardship. Table 2 summarizes comparisons of prevalence of financial hardship by cancer survivorship status and age group. We also summarize financial hardship prevalence estimates for the overall study sample in Supplementary Table 2. Overall, across age groups certain demographic groups of cancer survivors experienced high (significantly greater than overall prevalence i.e., < 58% in 18–59 & < 33% in 60+) burden of financial hardship. Among 18–59 cancer survivors, those who were never married (85%), NH-Black (82%), unemployed due to COVID-19 or unable to find employment (81%), those without a high school diploma (93%) or high school graduates (92%), those with a household income < $30,000 (91%), those living in rural areas (68%), and with an existing mental health condition (76%) had a high prevalence of financial hardship. Cancer survivors aged 18–59 on Medicaid (91%), Medicare (84%), dually insured with Medicaid + Medicare (95%), insured through the Indian Health Service (100%) or uninsured (87%) also had a high burden of financial hardship.

**Table 2:** Prevalence of financial hardship overall and among cancer survivors stratified by age groups among COVID Impact Survey respondents (*n* = 10,760), a nationally representative survey of the US (April-June 2020).

	**Total (*n =* 10,760)**				Cancer survivors (*n =* 854)^*^			
	**18–59 years old**		**60**+ **years**		**18–59 years old**		**60**+ **years**	
	**Row %**	**95% CI**	**Row %**	**95% CI**	**Row %**	**95% CI**	**Row %**	**95% CI**
Overall prevalence	48.9		32.4		57.6		33.2	
**Sex**								
Male	44.0	41.6, 46.4	27.5	24.8, 30.5	52.8	41.5, 63.9	25.6	19.9, 32.2
Female	53.4	51.3, 55.6	36.9	33.9, 40.0	60.8	50.6, 70.1	41.4	33.6, 49.6
**Marital status**								
Married/living with partner	42.3	40.3, 44.4	24.2	21.7, 26.9	44.5	34.9, 54.4	29.2	22.7, 36.7
Widowed/divorced/separated	61.8	57.6, 65.8	43.2	39.4, 47.1	64.5	49.6, 77.0	42.8	34.4, 51.6
Never married	56.3	53.1, 59.4	42.0	35.0, 49.4	84.8	71.6, 92.5	22.8	12.7, 37.4
**Race/ethnicity**								
White, NH	42.0	40.0, 44.0	25.8	23.6, 28.0	53.2	44.1, 62.1	26.3	21.4, 31.9
Black, NH	67.4	63.3, 71.3	64.6	57.7, 71.0	81.7	57.4, 93.7	79.2	61.7, 90.1
Hispanic	63.9	59.9, 67.8	37.3	29.8, 45.6	62.2	39.4, 80.6	17.4	8.5, 32.4
Asian, NH	31.3	24.3, 39.2	35.6	19.9, 55.2	63.6	28.7, 88.3	100.0	
Other	44.5	37.8, 51.4	42.8	32.3, 53.9	34.4	10.8, 69.5	45.6	28.0, 64.3
**Employment status in the past 7 days**								
Employed in the last 7 days	40.0	38.0, 42.0	29.4	25.4, 33.8	45.8	36.2, 55.8	28.0	19.7, 38.1
Retired/not interested in working at this time	54.1	49.5, 58.6	30.8	28.3, 33.4	62.7	45.5, 77.1	33.9	28.0, 40.3
Unemployed due to COVID-19 or unable to find employment^†^	66.6	63.4, 69.6	50.8	43.6, 58.0	81.3	67.0, 90.3	36.8	21.0, 56.1
**Education**								
No HS diploma	75.9	70.0, 80.9	59.6	48.4, 69.9	93.0	74.2, 98.4	76.0	53.6, 89.7
Hs graduate	60.7	57.1, 64.3	40.4	36.0, 44.9	92.0	82.5, 96.6	41.1	30.7, 52.3
Some college	52.1	49.8, 54.4	34.0	31.0, 37.1	49.2	37.9, 60.6	32.0	25.6, 39.1
Baccalaureate or above	27.8	25.6, 30.0	19.2	16.6, 22.2	30.9	20.6, 43.5	20.0	13.8, 28.3
**Household income**								
< $30,000	70.3	66.9, 73.4	55.6	51.1, 59.9	91.0	81.9, 95.8	59.6	49.0, 69.4
$30,000- < $50,000	59.4	55.8, 62.9	35.0	30.7, 39.6	61.4	44.7, 75.8	34.1	24.8, 45.0
$50,000- < $75,000	44.0	40.5, 47.6	24.8	20.7, 29.4	50.7	33.8, 67.5	22.5	13.7, 34.8
$75,000- < $100,000	37.3	33.4, 41.4	21.5	16.6, 27.4	39.7	21.8, 60.7	19.1	10.1, 33.3
≥$100,000	27.2	24.2, 30.6	11.2	8.8, 14.3	40.7	27.9, 54.9	9.5	5.1, 17.0
**Region**								
Northeast	45.3	41.0, 49.6	35.5	30.1, 41.3	63.1	43.4, 79.2	31.0	19.7, 45.3
Midwest	44.8	41.7, 47.9	30.4	26.4, 34.6	56.3	41.5, 70.1	29.9	21.2, 40.3
South	52.7	49.9, 55.4	33.9	30.4, 37.6	56.9	43.2, 69.5	39.2	30.3, 48.7
West	49.0	45.8, 52.2	29.4	25.6, 33.4	55.8	41.9, 68.9	27.8	20.0, 37.2
**Population density**								
Rural	49.1	43.8, 54.3	38.9	32.3, 45.9	67.5	44.8, 84.1	51.2	35.8, 66.4
Suburban	50.6	47.1, 54.2	25.1	21.4, 29.1	50.0	35.0, 65.0	18.6	11.6, 28.7
Urban	48.4	46.5, 50.4	33.7	31.1, 36.3	58.9	49.5, 67.7	32.9	27.2, 39.0
**Comorbid conditions**								
Cardiometabolic diseases^‡^	55.3	52.3, 58.3	36.5	33.8, 39.3	59.8	48.0, 70.5	37.4	31.1, 44.0
Respiratory diseases^§^	58.0	54.7, 61.2	39.2	34.8, 43.8	69.1	57.0, 79.1	41.7	32.0, 52.1
Overweight/obesity	54.6	51.9, 57.2	37.7	34.1, 41.4	60.9	49.5, 71.2	36.5	28.3, 45.5
Mental health conditions	59.8	56.2, 63.3	46.3	38.6, 54.1	76.3	59.6, 87.5	47.6	30.4, 65.3
**Insurance type or health coverage plans**								
Purchased plan	52.6	47.6, 57.6	28.5	24.8, 32.5	55.5	33.0, 75.9	26.4	18.5, 36.0
Employer-sponsored	37.0	35.0, 39.1	28.2	24.9, 31.8	45.1	35.8, 54.8	28.4	21.3, 36.7
Tricare	41.8	34.9, 49.0	20.6	15.2, 27.4	37.3	12.6, 70.9	19.9	8.9, 38.6
Medicaid	74.9	72.0, 77.7	51.6	47.0, 56.1	91.4	82.7, 95.9	52.3	41.7, 62.7
Medicare	66.6	59.9, 72.6	31.6	29.1, 34.2	83.9	68.3, 92.7	34.1	28.4, 40.3
Dually eligible (medicare and medicaid)	74.8	68.1, 80.5	50.9	45.9, 55.9	95.2	83.6, 98.7	55.5	44.0, 66.3
VA	39.9	32.0, 48.4	31.3	24.8, 38.6	26.4	7.8, 60.3	30.6	18.4, 46.2
Indian health service	73.9	60.2, 84.1	32.1	9.8, 67.3	100.0		0.0	
No insurance	67.4	62.6, 71.9	44.2	32.0, 57.2	86.6	57.6, 96.8	58.2	21.2, 87.8

* 2.46% of participants either chose: not sure, skipped or refused, when asked about their chronic conditions including cancer.

† Response Options: I was laid-off temporarily or furloughed, I was not at my usual jobs because I was caring for children not in school, I was not at my usual jobs because I was caring for an elderly person, I was not at my usual jobs because I was caring for someone with COVID-19, I was not at my usual jobs because I was recovering from COVID-19 or isolating due to exposure to COVID-19, I was unemployed but looking for work since March 1st, 2020 when COVID-19 began spreading in the US, I was unemployed and began looking for work after March 1, 2020 when COVID-19 began spreading in the US.

‡ Cardiometabolic conditions: diabetes, high blood pressure, heart disease, liver disease or end stage liver disease.

§ Respiratory conditions: Asthma, chronic lung disease or COPD, bronchitis, or emphysema.

Among cancer survivors aged 60 + years, we observed a similar trend in terms of key demographics with a higher burden of financial hardship. Women (41%), those who are widowed, divorced, or separated (43%), NH-Black (79.2%), NH-Asian (100%), without a high school diploma (76%), with a household income < $30,000 (60%), and living in rural areas (51%) who were 60+ cancer survivors had a high burden of financial hardship. Cancer survivors over 60+ years of age with mental health conditions (48%), Medicaid insured (52%) or uninsured (58%) also had a high burden of financial hardship.

Table 3 summarizes associated characteristics of financial hardship among cancer survivors. In the overall model, compared to cancer survivors aged 60+ years, those aged 30–44 (aPR:1.74, 95% CI:1.35–2.24), and 45–59 years (aPR:1.60, 95% CI:1.27–1.99) were more likely to experience financial hardship. Adult cancer survivors below the age of 60 who were never married (aPR: 1.32; 95% CI: 1.01–1.73) and Medicare insured (aPR: 1.33; 95% CI: 1.04–1.71) or uninsured (aPR: 2.13; 95% CI: 1.23–3.71) were more likely to report financial hardship compared to their counterparts. Among older (60+) cancer survivors, women had a 35% higher prevalence of financial hardship compared to men (aPR: 1.35; 95% CI: 1.02–1.78). Racial disparities exist in financial hardship among older cancer survivors: Compared to NH-White cancer survivors, NH-Black (aPR: 1.80; 95% CI: 1.32–2.45) and NH-Asian (aPR: 10.70; 95% CI: 5.55–20.66) had higher prevalenceof financial hardship. Lower income in cancer survivors aged 60+ led to higher prevalence of financial hardship with those earning < $30,000 over three times the prevalence of financial hardship compared to those earning over $100,000 (aPR: 3.63; 95% CI: 1.74–7.57). Like younger cancer survivors, older cancer survivors insured through Medicaid were more likely to experience financial hardship (aPR: 1.45; 95% CI: 1.03–2.06). Higher educational level decreased the prevalence of financial hardship among cancer survivors aged 18–59 and 60+ years. Supplementary Table 3 summarizes the associated characteristics of financial hardship among the general population overall and stratified by age demonstrating similar trends in risk factors, particularly in the 60+ years age group, excluding associations with sex, suggesting that women with cancer may be a particularly vulnerable group to financial hardship. Supplementary Table 4 summarizes estimates using logistic regression and demonstrates similar results to our main findings.

**Table 3:** Associated characteristics of financial hardship among cancer survivors in the COVID Impact Survey, a nationally representative survey of US (*n* = 854) (April–June 2020).

	**Overall**				**18–59 years**				**60**+ **years**			
	**PR**	**95% CI**	**aPR**	**95% CI**	**PR**	**95% CI**	**aPR**	**95% CI**	**PR**	**95% CI**	**aPR**	**95% CI**
**Age**					–							
18–29	1.51	0.88–2.59	1.32	0.90–1.95								
30–44	2.24	1.79–2.81	1.74	1.35–2.24								
45–49	1.56	1.22–1.98	1.60	1.27–1.99								
60+	Ref.		Ref.									
**Sex**												
Male	Ref.		Ref.		Ref.		–		Ref.		Ref.	
Female	1.46	1.18–1.82	1.10	0.89–1.35	1.15	0.88–1.51			1.61	1.19–2.20	1.35	1.02–1.78
**Marital status**												
Married/Living with partner	Ref.		Ref.		Ref.		Ref.		Ref.		Ref.	
Widowed/divorced/separated	1.45	1.15–1.82	1.16	0.94–1.42	1.45	1.06–1.98	1.14	0.77–1.68	1.47	1.07–2.01	1.28	0.94–1.74
Never married	1.68	1.26–2.24	1.13	0.85–1.50	1.91	1.48–2.46	1.32	1.01–1.73	0.78	0.43–1.42	0.86	0.46–1.60
**Race/ethnicity**												
White, NH	Ref.		Ref.		Ref.		Ref.		Ref.		Ref.	
Black, NH	1.89	1.21–2.94	1.56	1.23–1.97	1.54	1.16–2.03	1.36	0.90–1.07	3.01	2.30–3.93	1.80	1.32–2.45
Hispanic	0.92	0.44–1.94	0.89	0.64–1.24	1.17	0.79–1.73	1.07	0.77–1.50	0.66	0.33–1.34	0.58	0.27–1.22
Asian, NH	1.23	0.36–4.21	1.81	0.79–4.14	1.19	0.68–2.10	1.29	0.77–2.18	3.80	3.11–4.64	10.70	5.55–20.66
Other, NH	1.37	0.79–2.37	1.29	0.97–1.72	0.65	0.24–1.73	0.59	0.34–1.02	1.73	1.09–2.75	1.16	0.79–1.69
Insurance type^*^												
Purchased plan	0.70	0.52–0.94	1.05	0.76–1.45	0.95	0.61–1.47	–		0.72	0.49–1.06	1.45	0.94–2.22
Employer–sponsored	0.77	0.62–0.96	1.38	0.98–1.93	0.58	0.45–0.74	1.19	0.76–1.85	0.78	0.56–1.08	–	
Tricare	0.49	0.26–0.94	0.61	0.30.1.29	0.62	0.25–1.54	–		0.58	0.27–1.25	–	
Medicaid	2.19	1.80–2.66	1.61	1.19–2.18	2.15	1.71–2.70	1.48	0.94–2.32	2.14	1.59–2.89	1.45	1.03–2.06
Medicare	0.91	0.73–1.12	–		1.63	1.30–2.03	1.33	1.04–1.71	1.14	0.78–1.66	–	
VA	0.69	0.44–1.08	0.87	0.54–1.39	0.45	0.15–1.32	–		0.89	0.54–1.46	–	
No insurance	1.73	1.20–2.51	1.63	0.99–2.68	1.53	1.19–1.97	2.13	1.23–3.71	1.79	0.88–3.62	–	
**Any comorbid conditions**	1.53	1.13–2.06	1.09	0.84–1.41	1.24	0.86–1.78	–		1.77	1.16–2.69	1.29	0.81–2.06
**Employment status**												
Not employed	Ref.		–		Ref.		Ref.		Ref.		–	
Employed/self–employed	0.93	0.74–1.17			0.62	0.48–0.80	0.98	0.75–1.29	0.83	0.57–1.21		
**Education**												
No HS diploma	Ref.		Ref.		Ref.		Ref		Ref.		Ref.	
Hs graduate	0.68	0.56–0.84	1.03	0.77–1.39	0.99	0.87–1.13	1.43	0.99–1.91	0.54	0.38–0.77	0.78	0.51–1.19
Some college	0.45	0,37–0.55	0.79	0.51–0.96	0.53	0.41–0.69	0.76	0.56–1.04	0.42	0.30–0.58	0.83	0.56–1.22
Baccalaureate or above	0.29	0.21–0.39	0.52	0.35–0.77	0.33	0.22–0.49	0.61	0.38–0.97	0.26	0.17–0.41	0.49	0.28–0.88
**Household income**												
< $30,000	2.67	1.87–3.80	1.59	1.10–2.28	2.24	1.58–3.17	1.03	0.73–1.45	6.26	3.34–11.75	3.63	1.74–7.57
$30,000– < $50,000	1.63	1.09–2.43	1.18	0.83–1.69	1.51	0.98–2.33	0.96	0.62–1.48	3.59	1.83–7.04	2.61	1.26–5.42
$50,000– < $75,000	1.17	0.74–1.84	1.04	0.72–1.51	1.25	0.76–2.03	0.87	0.58–1.30	2.37	1.10–5.10	1.78	0.82–3.86
$75,000– < $100,000	0.96	0.57–1.62	1.09	0.72–1.67	0.97	0.52–1.81	0.87	0.53–1.44	2.01	0.85–4.74	1.93	0.88–4.26
≥$100,000	Ref.		Ref.		Ref.		Ref.		Ref.		Ref.	
**Region**												
Northeast	Ref.		–		Ref.		–		Ref.		–	
Midwest	0.91	0.64–1.30			0.89	0.60–1.33			0.96	0.57–1.64		
South	1.01	0.73–1.41			0.90	0.62–1.32			1.26	0.78–2.05		
West	0.91	0.64–1.28			0.88	0.60–1.30			0.90	0.53–1.51		
**Population density**												
Rural	1.27	0.97–1.68	0.99	0.77–1.27	1.14	0.81–1.62	–		1.56	1.09–2.23	1.09	0.75–1.60
Suburban	0.72	0.53–0.98	0.76	0.58–0.99	0.85	0.60–1.20			0.57	0.35–0.93	0.71	0.41–1.22
Urban	Ref.		Ref.		Ref.				Ref.		Ref.	

PR, Unadjusted prevalence ratio; aPR, Adjusted prevalence ratio; CI, Confidence intervals; Ref, Reference.

–: This variable was not included in the final full model due to specifications outlined in the method section (p < 0.10).

* Insurance variables modeled as binary (i.e., those with the specific insurance type vs. not).

All models are adjusted for survey week.

### Mental health and financial hardship among cancer survivors

**Figure 1:**
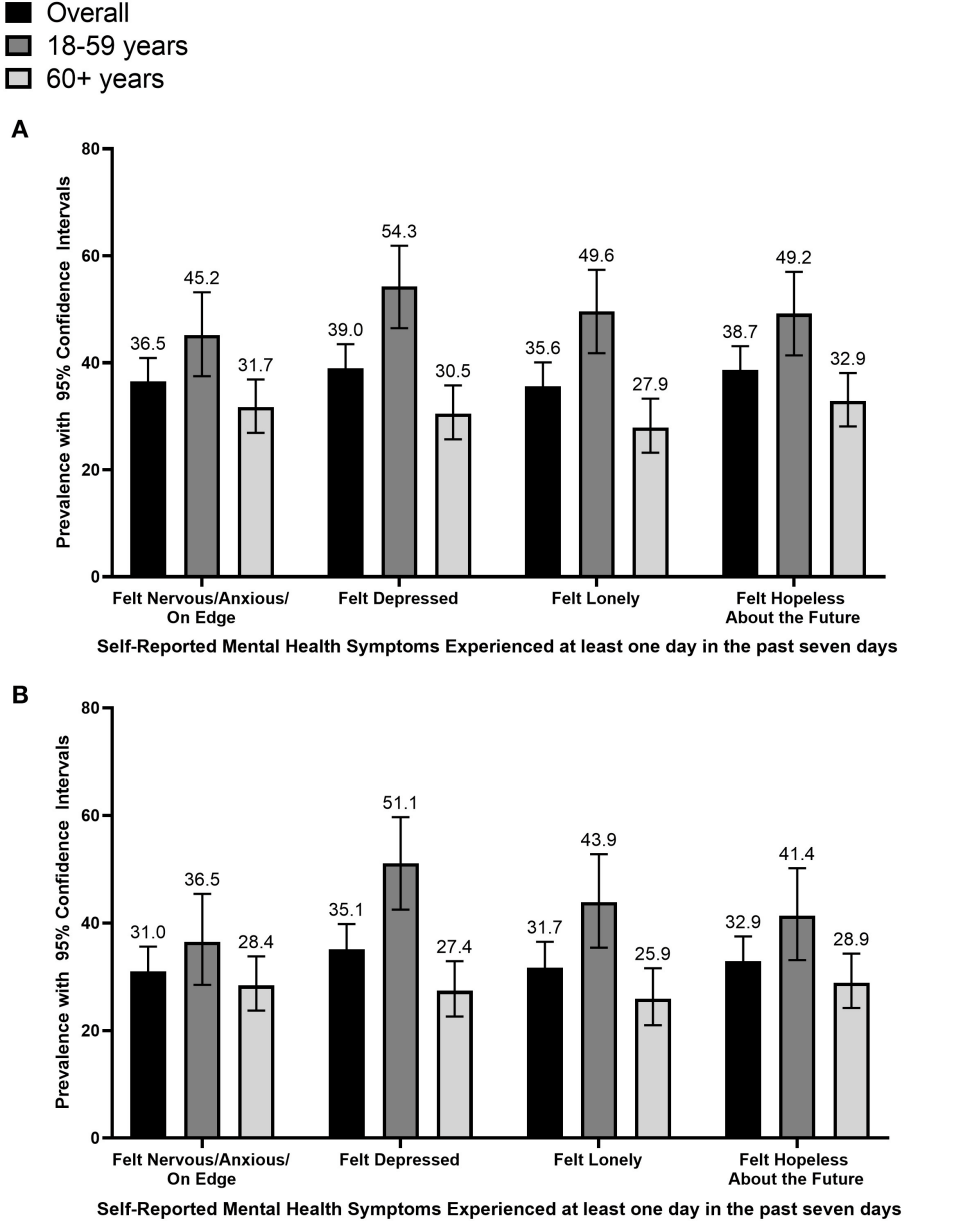
Prevalence of mental health symptoms among cancer survivors stratified by age group, COVID-19 Household Impact Survey (April–June 2020) (*n* = 854). This figure summarizes the prevalence of mental health symptoms experienced at least one time in the past seven days, specifically among **(A)** all cancer survivors and **(B)** cancer survivors without a diagnosed mental health condition.

Figure 1 summarizes the prevalence of mental health symptoms at least 1 day a week of financial hardship with mental health symptoms among cancer survivors. Cancer survivors 18–59 years were more likely to report feeling anxious (45 vs. 32%, *p* = 0.004), depressed (54 vs. 31%, *p* < 0.001), lonely (50 vs. 28%, *p* < 0.001), and hopeless about the future (49% vs. 33%, *p* = 0.001). Among cancer survivors without a self-reported diagnosed mental health condition, we observed similar differences across age group. Table 4 summarizes the associations of financial hardship with mental health symptoms overall and stratified by age group. Overall, among cancer patients without a history of mental health conditions, financial hardship was associated with feelings of anxiety (aPR:1.51; 95% CI:1.11–2.05), depression (aPR:1.66; 95%CI:1.25–2.22), and hopelessness about the future (aPR:1.84; 95% CI:1.38–2.44). Specifically, among 18–59 cancer survivors, financial hardship was associated with feelings of depression (aPR: 2.09; 95% CI: 1.45–3.02). And among 60+ cancer survivors, financial hardship was associated with feeling hopeless about the future (aPR: 1.76; 95% CI: 1.19–2.59). Supplementary Table 5 summarizes our estimates using logistic regression and demonstrates the same findings as our main analyses.

**Table 4:** Associations of financial hardship with mental health symptoms experienced at least 1 day in the past week among cancer survivors by age groups among COVID Impact Survey cancer survivors, a nationally representative survey of the US (April–June 2020).

	**Overall**			**18–59 years**			**60**+ **years**		
**All cancer patients (*n* = 854)**	**aPR**	**95% CI**		**aPR**	**95% CI**		**aPR**	**95% CI**	
Felt nervous, anxious, on edge	1.76	1.37	2.25	1.63	1.13	2.35	1.58	1.11	2.26
Felt depressed	1.83	1.44	2.31	2.00	1.46	2.75	1.24	0.87	1.77
Felt lonely	1.48	1.18	1.84	1.13	0.83	1.54	1.38	1.00	1.91
Felt hopeless about the future	1.95	1.53	2.47	1.42	0.99	2.04	1.73	1.22	2.46
	**Overall**			**18–59 years**			**60**+ **years**		
**Cancer patients without a self–reported diagnosed mental health condition (*****n*** **= 737)**	**aPR**	**95% CI**		**aPR**	**95% CI**		**aPR**	**95% CI**	
Felt nervous, anxious, on edge	1.51	1.11	2.05	1.38	0.84	2.28	1.36	0.89	2.10
Felt depressed	1.66	1.25	2.22	2.09	1.45	3.02	1.02	0.66	1.57
Felt lonely	1.31	0.99	1.73	1.12	0.75	1.66	1.21	0.81	1.80
Felt hopeless about the future	1.84	1.38	2.44	1.23	0.80	1.89	1.76	1.19	2.59

Models were adjusted for: age (when appropriate), survey week, sex, race/ethnicity, annual household income, education, insurance status, employment status, and area of residence (urban/rural).

## Discussion

Overall, our study demonstrated that over four in 10 cancer survivors reported financial hardship during the COVID-19 pandemic in the United States. Cancer survivors of younger age groups (< 60 years), lower educational attainment, lower income, Medicaid-insured, and racial/ethnic minoritized cancer survivors had a higher burden of financial hardship during the COVID-19 pandemic. Financial hardship was associated with mental health symptoms, including depression, anxiety, and hopelessness, among cancer survivors, even amongst those without an existing mental health condition. Findings from our analyses are consistent with prior studies focused on financial hardship among cancer survivors (28, 31, 38, 39). Our analyses underscore the potential impact of the pandemic on mental health given that associations between adverse mental health symptoms was associated with financial hardship, even amongst those without a history of a mental health condition.

In our study we found that financial hardship among cancer survivors is most common among those of younger age, racial/ethnic minoritized communities, and those with markers of lower socioeconomic status, including Medicaid insurance, lower income, and lower educational attainment. Our findings are consistent with research focused on financial hardship among cancer survivors prior to the pandemic, which has shown that certain demographic groups are more vulnerable to financial toxicity, a phenomenon coined to underscore the detrimental impact of the costs of cancer care in the U.S. (40). Sociodemographic associated characteristics of financial toxicity among cancer patients identified prior to the pandemic include female sex, non-partnered marital status, Black and Hispanic race and ethnicity, low income, loss of income, younger age, and being uninsured (41, 42). While we observed a similar prevalence in financial hardship across cancer survivorship status among all ages, younger (18–59 years) cancer survivors experienced a significantly higher burden of financial hardship compared to their counterparts in the total population. In fact, one in five younger cancer survivors reported they would not be able to cover a sudden $400 expense based on their current situation. Younger age (< 65 years) has been positively associated with financial hardship among cancer patients, including a dose-response relationship across the age spectrum (43). Younger cancer survivors, particularly those of working age, may be more likely to report financial hardship due to employment interruptions or reduced hours due to limitations in the ability to work leading to potential concerns regarding health insurance coverage (44). In fact, we observed that one-quarter of cancer survivors aged 18–59 reported they were either under- or unemployed due to COVID-19 or unable to find employment; Financial hardship was very high amongst this group with 81% experiencing economic precarity. As employment is closely tied to health insurance coverage in the U.S., these cancer survivors are particularly vulnerable to toxic financial shocks associated with both active and survivorship care even after their cancer may be in remission. Older adults over the age 65 years are covered through Medicare, and other benefits such as Social Security, which may alleviate the financial burdens associated with a cancer diagnosis. In addition to younger age, lower income and lower education have been associated with financial hardship among cancer survivors (44–48). Prior research has also demonstrated Medicare (49, 50), supplemental (51), and commercial insurance (52) coverage are associated with decreased financial burdens compared with patients covered with Medicaid. Insurance coverage plays a pivotal role in financial hardships among cancer survivors due to the associated out-of-pocket spending based on type of insurance plan, premiums, and deductibles.

Racial/ethnic disparities in financial toxicity among cancer survivors have been previously demonstrated, with NH-Black or African American and Hispanic/Latinx cancer patients frequently cited as experiencing higher odds of financial toxicity (41, 42). In our study population of cancer survivors, racial/ethnic inequities in financial hardship were particularly prominent among cancer survivors aged 60+ years as demonstrated in Table 3. We observed that NH-Black or African American cancer survivors as well as NH-Asian American cancer survivors had the highest burden of financial hardship. While our sample of Asian American cancer survivors was small, it is a striking finding and should be explored further given the novelty of this finding. Although limited prior work has evaluated financial toxicity among this group specifically, research focused on Asian American cancer patients suggests that cost of cancer treatment and care contribute to poor outcomes in this demographic group (53). Qualitative work conducted among Chinese American cancer patients residing in an urban area of California suggest that up to 80%, or a large majority, were interested in culturally-tailored educational programs regarding financial and social assistance during their cancer treatment (53). Another survey of Asian American cancer patients to identify their unmet needs during cancer treatment found that almost one-third of respondents indicated they have difficulties meeting basic living expenses and almost half reported they have some type of financial difficulty (54). Future research to further investigate the financial wellbeing of Asian American cancer patients, and the population in general, should be prioritized, particularly after the COVID-19 pandemic during the rise of anti-Asian hate in the United States and the associated job security experienced by this group (55).

Financial hardship was measured in the COVID-19 Impact Survey using a question developed by the US Federal Reserve and included in the Economic Wellbeing of U.S. Households (SHED) survey, which is implemented to “share the wide range of financial challenges and opportunities facing individuals and households in the United States (56).” In 2018, the latest year of data available, the SHED survey results suggested that 40% of adults would experience hardship covering a $400, which was a 2% point increase from the prior year (27). We similarly observed the prevalence of financial hardship was 44%. Our study was unable to specify whether financial hardships experienced by cancer survivors were directly due to costs associated with cancer treatment; however, cancer is a chronic disease that often involves long-term adjuvant care and management of long-term adverse effects that continue to elevate medical costs over the patient's life (57). The inability to cover an unexpected $400 expense, however, is telling of the financial precarity of cancer survivors during the COVID-19 pandemic (58). In our definition of financial hardship, we included those who reported they would have to resort to one of the following options: put it on my credit card and pay if off over time; use money from a bank loan or line of credit; I wouldn't be able to pay for it right now; sell something; use a payday loan, deposit advance or overdraft; borrow from a friend or family member. Given the high costs associated with cancer survivorship care, these options for covering a sudden $400 expense may be unsustainable and suggests that additional health care associated costs may not be prioritized. Based on data from SEER-Medicare, average cancer survivorship annualized costs for those aged 65 years or above can range from $5,300–105,000 for medical care and $1,100–4,200 for oral prescription drugs depending on the phase across the cancer care continuum (59). Based on a 2018 review, annual out-of-pocket costs to recently diagnosed cancer survivors were more than $1,000 for medical care and time costs (i.e., patient time associated with cancer treatment such as round-trip travel time, waiting for care, and receiving care), approximately $2,000 for productivity losses, and from $2,500 to >$4,000 for employment disability, depending on age. For longer term survivors, the cost of medical care was approximately $1,500 for older survivors and $747 for younger survivors, time costs ranged from $831- $955 for older survivors and $459-$630 for younger survivors, and productivity losses were approximately $800 (12). Strategies to mitigate the financial hardships experienced by cancer survivors, particularly in the context of the negative economic downstream effects of COVID-19 in the US (20, 60), should be prioritized.

The COVID-19 pandemic has led to increased mental health symptoms among cancer survivors, even among those without an existing mental health condition (61). Reports from early in the pandemic demonstrate that US cancer survivors are more likely to report frequently feeling nervous anxious or on edge, depressed, lonely, and hopeless during the week, particularly those with limited social interaction with friends or family (61). Similarly, among older breast cancer survivors in the US, increased loneliness during the COVID-19 pandemic was associated with worsening depression symptoms and higher stress (62). Stressors contributing to poor mental health outcomes among cancer survivors during the pandemic include uncertainty regarding future cancer care, fears about in-person appointments, cancer recurrence due to care delays, and distress about untreated symptoms including mental health issues (63). Indeed, cancer survivors during the COVID-19 pandemic resorted to canceling or delaying care (26), which presents barriers to cancer survivor's ability to discuss their concerns and worries with their health care team. Our study demonstrates the potential role of financial stressors, including financial hardship, on the mental health outcomes of cancer survivors during the COVID-19 pandemic. As the COVID-19 pandemic continues to disproportionately impact patients with chronic conditions including cancer survivors, providers or care teams may consider prioritizing conversations or assessments of mental health during opportunistic care visits.

There are several limitations that should be considered when interpreting the results of our analyses. First, data leveraged for this analysis are cross-sectional in nature and may lead to reverse causality when evaluating associations of mental health with financial hardship. Second, our main outcome of financial hardship was based on a questionnaire item that has not been previously used in studies evaluating financial hardship or toxicity among cancer survivors. The questionnaire is generally used in economic US surveys such as the annual Survey of Household Economics and Decision-making (SHED) (64). We were unable to assess if the survey respondent's financial situation has changed since the COVID-19 pandemic began or were these financial constrains already in existence. Further, data collection occurred early in the pandemic period (April–June 2020), however, financial hardship and mental health may have worsened in later periods of the pandemic given the persistent adverse economic impact of the pandemic. Next, the definition of our study population of cancer survivors was based on self-report leading to the potential for measurement error in our definition of a cancer survivor. Similarly, we relied on self-report of mental health symptoms reported in the seven days before survey administration. Data on psychological distress measured using validated scales, such as the General Anxiety Disorder-7 (GAD-7), were not available. We were unable to measure and account for important cancer-related variables such as cancer site, stage, time since diagnosis, type of treatment (surgery/chemo/radiation), and whether the respondents were currently in active treatment. Patients with very aggressive cancers and potentially expensive treatment may have been unlikely to be reached or may not have survived long enough to be part of the survey; thus, our analysis may underestimate financial hardship and its impact. Further it is important to note that about one in five adults offered the survey provided a response, which may have led to a non-response bias given those who are experiencing adverse social issues may be less likely to respond. Nevertheless, a notable strength of our analysis is we utilized nationally representative survey data and therefore, obtained a representative sample of cancer survivors in the US. Through this analysis we were able to provide preliminary insights into the financial impact of cancer survivors in the U.S. during the COVID-19 pandemic and potentially identify demographic groups that may be most affected.

In conclusion, our study demonstrates that four out of ten cancer survivors are experiencing financial hardship during the COVID-19 pandemic, with the most vulnerable being younger adults, those with low income, racial/ethnic minorities, and the Medicaid-insured. Given the negative impact the COVID-19 pandemic has had on the US economy, considering financial strife in the context of cancer survivorship care should be prioritized among US oncologists when discussing future care plans. Additionally, plans to alleviate or address mental health outcomes among cancer survivors during the pandemic should also be addressed. As poor mental health outcomes have been associated with adverse health consequences including forgoing or delaying necessary medical health, as well as poor adherence to treatment (17), strategies to address cost barriers to accessing high-quality survivorship care are needed to alleviate the negative impacts on quality of life of cancer survivors.

## Data availability statement

Publicly available datasets were analyzed in this study. This data can be found here: https://www.covid-impact.org/.

## Ethics statement

Ethical review and approval was not required for the study on human participants in accordance with the local legislation and institutional requirements. Written informed consent for participation was not required for this study in accordance with the national legislation and the institutional requirements.

## Author contributions

JI and MC-R conceived the study idea, developed the analysis plan, and drafted the first version of the manuscript. JI conducted all data analyses. All authors contributed to the interpretation of results and the final version of the manuscript. All authors contributed to the article and approved the submitted version.

## Conflict of interest

The authors declare that the research was conducted in the absence of any commercial or financial relationships that could be construed as a potential conflict of interest.

## Publisher's note

All claims expressed in this article are solely those of the authors and do not necessarily represent those of their affiliated organizations, or those of the publisher, the editors and the reviewers. Any product that may be evaluated in this article, or claim that may be made by its manufacturer, is not guaranteed or endorsed by the publisher.
