# Supplementary material for: Financial hardship and mental health among cancer survivors during the COVID-19 pandemic: An analysis of the US COVID-19 Household Impact Survey
Source: Front Public Health. 2022 Nov 22;10:946721. doi: 10.3389/fpubh.2022.946721 (PMC9723235; doi:10.3389/fpubh.2022.946721)
Supplement: Supplementary file 1 [file Data_Sheet_1.docx]

| Supplementary Table 1: Characteristics of COVID Impact Survey respondents (n = 10,760), a nationally representative survey of the US (April-June 2020) | | | | |
| --- | --- | --- | --- | --- |
|  | Total | | Cancer Survivors | |
|  | (n=10, 760) | | (n=854) | |
|  | Col % | 95% CI | Col % | 95% CI |
| Age |  |  |  |  |
| 18-29 | 20.5 | 19.3,21.9 | 3 | 1.8,4.9 |
| 30-44 | 25.3 | 24.2,26.4 | 9.4 | 6.9,12.6 |
| 45-59 | 24.3 | 23.2,25.4 | 23 | 19.4,27.0 |
| 60+ | 29.9 | 28.8,31.1 | 64.7 | 60.3,68.9 |
| Sex |  |  |  |  |
| Male | 48.4 | 47.0,49.7 | 47.6 | 43.2,51.9 |
| Female | 51.6 | 50.3,53.0 | 52.4 | 48.1,56.8 |
| Marital Status |  |  |  |  |
| Married/Living with Partner | 57.3 | 55.9, 58.6 | 57 | 52.6, 61.3 |
| Widowed/Divorced/Separated | 18.5 | 17.5, 19.5 | 31.2 | 27.3, 35.4 |
| Never Married | 24.2 | 23.0, 25.5 | 11.8 | 9.1, 15.3 |
| Race/Ethnicity |  |  |  |  |
| White, NH | 62.1 | 60.8,63.4 | 74.3 | 70.0,78.1 |
| Black, NH | 11.6 | 10.8,12.4 | 11.6 | 8.7,15.4 |
| Hispanic | 16.4 | 15.3,17.5 | 7.8 | 5.6,10.6 |
| Other, NH | 9.9 | 9.1,10.8 | 6.3 | 4.7,8.5 |
| Employed in the past 7 days | 50 | 48.6,51.3 | 32 | 28.1,36.2 |
| Education |  |  |  |  |
| No HS Diploma | 9.6 | 8.7,10.7 | 6.4 | 4.4,9.3 |
| HS Graduate | 28.1 | 26.8,29.5 | 30.3 | 26.0,34.9 |
| Some College | 27.7 | 26.7,28.8 | 27.9 | 24.7,31.4 |
| Baccalaureate or Above | 34.5 | 33.3,35.7 | 35.4 | 31.3,39.7 |
| Household Income |  |  |  |  |
| <$30,000 | 26.8 | 25.6,28.1 | 26.9 | 23.0,31.2 |
| $30,000-<$50,000 | 18.7 | 17.8,19.7 | 21.6 | 18.3,25.4 |
| $50,000-<$75,000 | 18.6 | 17.6,19.6 | 17 | 14.1,20.4 |
| $75,000-<$100,000 | 13.5 | 12.7,14.5 | 9.9 | 7.8,12.5 |
| ≥$100,000 | 22.4 | 21.3,23.5 | 24.5 | 20.9,28.5 |
| Region |  |  |  |  |
| Northeast | 17.4 | 16.3,18.5 | 17.2 | 13.9,21.1 |
| Midwest | 20.8 | 19.9,21.8 | 22.4 | 19.1,26.1 |
| South | 37.9 | 36.5,39.2 | 34.9 | 30.7,39.2 |
| West | 23.9 | 22.9,25.1 | 25.5 | 22.0,29.4 |
| Population Density |  |  |  |  |
| Rural | 9 | 8.3,9.8 | 13.4 | 10.4,17.0 |
| Suburban | 18.7 | 17.7,19.7 | 20 | 16.9,23.6 |
| Urban | 72.3 | 71.1,73.4 | 66.6 | 62.3,70.7 |
| Comorbid Conditions |  |  |  |  |
| Cardiovascular disease | 37.8 | 36.5,39.1 | 60.2 | 55.8,64.4 |
| Respiratory diseases | 23.5 | 22.4,24.7 | 29.3 | 25.4,33.5 |
| Overweight/Obesity | 33.3 | 32.1,34.5 | 41.6 | 37.3,46.0 |
| Mental Health Conditions | 15.3 | 14.4,16.3 | 13.3 | 10.5,16.7 |
|  |  |  |  |  |
| Insurance Type or Health Coverage Plans |  |  |  |  |
| Purchased Plan | 17.1 | 16.1,18.2 | 20.2 | 17.0,23.8 |
| Employer-Sponsored | 51.9 | 50.6,53.3 | 47.0 | 42.6,51.4 |
| TRICARE | 4.8 | 4.3,5.4 | 6.8 | 4.8,9.5 |
| Medicaid | 23.5 | 22.3,24.6 | 30.3 | 26.1,34.8 |
| Medicare | 25.4 | 24.3,26.6 | 56.1 | 51.6,60.4 |
| Dually Eligible (Medicare & Medicaid) | 10.1 | 8.9,11.3 | 9.0 | 7.9,10.3 |
| VA | 4.4 | 4.0,4.9 | 8.8 | 6.6,11.6 |
| Indian Health Service | 1.2 | 0.9,1.6 | 0.3 | 0.1,0.8 |
| No insurance | 8.8 | 8.0,9.6 | 3.0 | 1.8,4.9 |
|  |  |  |  |  |
| Financial Hardship Measure | |  |  |  |
| Suppose that you have an unexpected expense that costs $400. Based on your current financial situation, how would you pay for this expense? If you would use more than one method to cover this expense, please select all that apply |  |  |  |  |
|  |  |  |  |  |
| Put it on my credit card and pay it off in full at the next statement | 34.2 | 33.0,35.5 | 39.4 | 35.3,43.7 |
| Put it on my credit card and pay it off over time | 18.6 | 17.6,19.6 | 17.8 | 14.8,21.2 |
| Use money currently in my checking or savings account or with cash | 51.1 | 49.7,52.4 | 52 | 47.6,56.4 |
| Use money from a bank loan or line of credit | 3 | 2.6,3.4 | 5.1 | 3.5,7.6 |
| Borrow from a friend or family member | 9.3 | 8.5,10.2 | 6.7 | 4.7,9.6 |
| Use a payday loan, deposit advance or overdraft | 1.9 | 1.6,2.3 | 1 | 0.5,2.0 |
| Sell something | 7 | 6.3,7.7 | 4.4 | 3.0,6.3 |
| I would not be able to pay for it right now | 15.4 | 14.4,16.5 | 13.9 | 10.6,17.9 |
| *2.46% of participants either chose not sure, skipped or refused, when asked about their chronic conditions including cancer | | | | |

| Supplementary Table 2: Prevalence of financial hardship overall and among cancer survivors among COVID Impact Survey respondents (n = 10,760), a nationally representative survey of the US (April-June 2020) | | | | |
| --- | --- | --- | --- | --- |
|  | Total | | Cancer Survivors | |
|  | (n=10, 760) | | (n=854) | |
|  | Row% | 95% CI | Row % | 95% CI |
|  | 44% |  | 42% |  |
| Age |  |  |  |  |
| 18-29 | 56.9 | 53.3,60.4 | 50.1 | 26.3,73.9 |
| 30-44 | 48.1 | 45.8,50.5 | 74.4 | 60.4,84.7 |
| 45-59 | 42.9 | 40.4,45.5 | 51.7 | 42.3,61.0 |
| 60+ | 32.4 | 30.3,34.6 | 33.2 | 28.2,38.5 |
|  |  |  |  |  |
| Sex |  |  |  |  |
| Male | 39.1 | 37.2,41.1 | 33.7 | 28.1,39.7 |
| Female | 48.5 | 46.7,50.3 | 49.2 | 42.9,55.6 |
|  |  |  |  |  |
| Marital Status |  |  |  |  |
| Married/Living with Partner | 37 | 35.4,38.7 | 34.3 | 28.8,40.2 |
| Widowed/Divorced/Separated | 51.5 | 48.6,54.3 | 49.6 | 41.9,57.3 |
| Never Married | 54.7 | 51.7,57.6 | 57.6 | 43.9,70.2 |
|  |  |  |  |  |
| Race/Ethnicity |  |  |  |  |
| White, NH | 36.4 | 34.9,38.0 | 35.3 | 30.6,40.3 |
| Black, NH | 66.6 | 63.1,70.0 | 80.1 | 66.5,89.0 |
| Hispanic | 59.3 | 55.6,62.9 | 41.1 | 25.9,58.3 |
| Asian, NH | 31.7 | 25.2,39.0 | 73.2 | 38.7,92.2 |
| Other | 44 | 38.3,49.9 | 43.3 | 27.9,60.2 |
|  |  |  |  |  |
| Employment Status in the Past 7 Days | |  |  |  |
| Employed in the last 7 days | 38.6 | 36.7,40.5 | 61.5 | 49.1,72.6 |
| Retired/Not interested in working at this time | 37.7 | 35.5,40.1 | 39.5 | 32.3,47.2 |
| Unemployed due to COVID-19 or unable to find employment † | 64.5 | 61.6,67.4 | 37.2 | 31.6,43.3 |
|  |  |  |  |  |
| Education |  |  |  |  |
| No HS Diploma | 72.5 | 67.2,77.1 | 83.8 | 69.4,92.2 |
| HS Graduate | 54.4 | 51.6,57.3 | 57.4 | 48.3,66.0 |
| Some College | 46.6 | 44.7,48.5 | 37.7 | 31.9,43.9 |
| Baccalaureate or Above | 25.1 | 23.4,26.9 | 24.2 | 18.4,31.1 |
|  |  |  |  |  |
| Household Income |  |  |  |  |
| <$30,000 | 65.8 | 63.1,68.4 | 69.3 | 60.8,76.6 |
| $30,000-<$50,000 | 50.8 | 47.9,53.6 | 42.4 | 33.6,51.7 |
| $50,000-<$75,000 | 38.8 | 35.9,41.7 | 30.3 | 21.8,40.5 |
| $75,000-<$100,000 | 32.7 | 29.5,36.1 | 25 | 16.4,36.2 |
| ≥$100,000 | 23 | 20.6,25.7 | 26 | 18.3,35.5 |
|  |  |  |  |  |
| Region |  |  |  |  |
| Northeast | 42.3 | 38.9,45.8 | 43.5 | 32.1,55.6 |
| Midwest | 40.4 | 37.9,42.9 | 39.7 | 31.4,48.7 |
| South | 47 | 44.8,49.3 | 44.1 | 36.6,51.9 |
| West | 43.5 | 40.9,46.1 | 39.4 | 31.6,47.8 |
|  |  |  |  |  |
| Population Density |  |  |  |  |
| Rural | 45.3 | 41.1,49.4 | 54.4 | 41.2,67.0 |
| Suburban | 42 | 39.2,44.8 | 30.7 | 22.8,39.8 |
| Urban | 44.3 | 42.8,45.9 | 42.7 | 37.3,48.1 |
|  |  |  |  |  |
| Comorbid Conditions |  |  |  |  |
| Cardiometabolic diseases‡ | 45.7 | 43.7,47.8 | 42.8 | 37.2,48.6 |
| Respiratory diseases§ | 52.2 | 49.5,54.9 | 53.9 | 45.9,61.7 |
| Overweight/Obesity | 49.2 | 47.0,51.4 | 46.2 | 39.3,53.3 |
| Mental Health Conditions | 57.9 | 54.6,61.1 | 64.1 | 52.0,74.7 |
|  |  |  |  |  |
| Insurance Type or Health Coverage Plans |  |  |  |  |
| Purchased Plan | 41 | 37.7,44.3 | 31.7 | 23.7,41.0 |
| Employer-Sponsored | 35.2 | 33.5,37.1 | 36.3 | 30.3,42.7 |
| TRICARE | 33.1 | 28.2,38.4 | 21.7 | 10.9,38.4 |
| Medicaid | 67.2 | 64.6,69.7 | 67.5 | 59.5,74.6 |
| Medicare | 37.1 | 34.8,39.5 | 40 | 34.4,45.9 |
| Dually Eligible (Medicare & Medicaid) | 58.8 | 54.8,62.8 | 65.5 | 56.0,73.8 |
| VA | 35.3 | 30.1,40.9 | 30.1 | 18.8,44.5 |
| Indian Health Service | 71.7 | 58.5,82.0 | 84.8 | 48.1,97.1 |
| No insurance | 65.4 | 60.8,69.7 | 71 | 41.9,89.2 |

| Supplementary Table 3: Associated Characteristics of financial hardship among all adults in the COVID Impact Survey, a nationally representative survey of US (n=854), (April-June 2020) | | | | | | | | | | | | | |
| --- | --- | --- | --- | --- | --- | --- | --- | --- | --- | --- | --- | --- | --- |
|  |  |  |  |  |  |  |  |  |  |  |  |  |  |
|  | Overall | | | | 18-59 years | | | | 60+ years | | | |  |
|  | PR | 95% CI | aPR | 95% CI | PR | 95% CI | aPR | 95% CI | PR | 95% CI | APR | 95% CI | |
| Overall Prevalnce | 43.90 |  |  |  | 48.90 |  |  |  | 32.40 |  |  |  | |
| Age |  |  |  |  |  |  |  |  |  |  |  |  | |
| 18-29 | 1.75 | 1.60-1.92 | 1.38 | 0.99-1.92 |  |  |  |  |  |  |  |  | |
| 30-44 | 1.48 | 1.37-1.61 | 1.91 | 1.42-2.56 |  |  |  |  |  |  |  |  | |
| 45-49 | 1.32 | 1.21-1.45 | 1.56 | 1.23-1.97 |  |  |  |  |  |  |  |  | |
| 60+ | Ref. |  | Ref. |  |  |  |  |  |  |  |  |  | |
| Sex |  |  |  |  |  |  |  |  |  |  |  |  | |
| Male |  |  |  |  |  |  |  |  |  |  |  |  | |
| Female | Ref. |  | Ref. |  | Ref. |  | Ref. |  | Ref. |  | Ref. |  | |
| Marital Status | 1.24 | 1.16-1.32 | 1.20 | 1.00-1.45 | 1.21 | 1.13-1.30 | 1.10 | 1.03-1.17 | 1.32 | 1.17-1.53 | 1.20 | 0.92-1.57 | |
| Married/Living  with Partner |  |  |  |  |  |  |  |  |  |  |  |  | |
| Widowed/Divorced  /Separated |  |  |  |  |  |  |  |  |  |  |  |  | |
| Never Married | Ref. |  | Ref. |  | Ref. |  | Ref. |  | Ref. |  | Ref. |  | |
| Race/Ethnicity | 1.39 | 1.30-1.49 | 1.17 | 0.96-1.42 | 1.46 | 1.35-1.59 | 1.20 | 1.11-1.30 | 1.78 | 1.55-2.05 | 1.24 | 0.901.71 | |
| White, NH | 1.48 | 1.38-1.59 | 1.05 | 0.84-1.32 | 1.33 | 1.24-1.43 | 1.10 | 1.02-1.18 | 1.74 | 1.42-2.13 | 0.82 | 0.46-1.47 | |
| Black, NH |  |  |  |  |  |  |  |  |  |  |  |  | |
| Hispanic |  |  |  |  |  |  |  |  |  |  |  |  | |
| Asian, NH | Ref. |  | Ref. |  | Ref. |  | Ref. |  | Ref. |  | Ref. |  | |
| Other, NH | 1.83 | 1.71-1.96 | 1.69 | 1.40-2.05 | 1.61 | 1.49-1.73 | 1.32 | 1.22-1.42 | 2.51 | 2.19-2.87 | 2.11 | 1.53-2.91 | |
| Insurance Type* | 1.63 | 1.51-1.76 | 1.05 | 0.80-1.38 | 1.52 | 1.41-1.65 | 1.23 | 1.13-1.34 | 1.45 | 1.15-1.82 | 0.62 | 0.31-1.23 | |
| Purchased Plan | 0.87 | 0.70-1.09 | 1.04 | 0.40-2.71 | 0.74 | 0.58-0.95 | 0.93 | 0.75-1.17 | 1.38 | 0.82-2.33 | 12.10 | 6.05-24.19 | |
| Employer-Sponsored | 1.21 | 1.05-1.39 | 1.27 | 0.96-1.68 | 1.06 | 0.90-1.25 | 0.92 | 0.80-1.07 | 1.66 | 1.27-2.18 | 1.16 | 0.79-1.67 | |
| TRICARE |  |  |  |  |  |  |  |  |  |  |  |  | |
| Medicaid |  |  |  |  |  |  |  |  |  |  |  |  | |
| Medicare | 0.91 | 0.83-0.99 | 1.01 | 0.76-1.35 | 1.08 | 0.97-1.19 | - |  | 0.84 | 0.72-0.98 | 0.97 | 0.62-1.51 | |
| VA | 0.66 | 0.62-0.70 | 1.48 | 1.10-2.01 | 0.56 | 0.53-0.60 | 0.94 | 0.85-1.04 | 0.81 | 0.70-0.94 | 1.79 | 1.12-2.84 | |
| No insurance | 0.74 | 0.63-0.86 | 0.52 | 0.27-1.01 | 0.84 | 0.71-1.00 | 0.98 | 0.83-1.17 | 0.62 | 0.46-0.84 | 0.57 | 0.27-1.18 | |
| Any Comorbid Conditions | 1.81 | 1.71-1.91 | 1.54 | 1.18-2.01 | 1.79 | 1.69-1.90 | 1.34 | 1.21-1.47 | 2.03 | 1.79-2.30 | 1.68 | 1.14-2.47 | |
| Employment Status | 0.80 | 0.74-0.86 | 1.18 | 0.91-1.54 | 1.38 | 1.25-1.53 | 0.97 | 0.87-1.08 | 0.94 | 0.81-1.08 | - |  | |
| Not Employed | 0.79 | 0.67-0.92 | 0.99 | 0.63-1.55 | 0.81 | 0.65-0.99 | 0.78 | 0.63-0.97 | 0.95 | 0.75-1.19 | - |  | |
| Employed/Self-Employed | 1.56 | 1.45-1.68 | 1.89 | 1.18-3.00 | 1.45 | 1.34-1.57 | 1.20 | 1.07-1.35 | 1.38 | 1.02-1.86 | 1.79 | 0.81-3.96 | |
| Education |  |  |  |  |  |  |  |  |  |  |  |  | |
| No HS Diploma | 1.58 | 1.18-2.12 | 1.19 | 0.92-1.54 | 1.26 | 0.89-1.80 | - |  | 1.74 | 1.15-2.64 | 1.18 | 0.76-1.83 | |
| HS Graduate |  |  |  |  |  |  |  |  |  |  |  |  | |
| Some College |  |  |  |  |  |  |  |  |  |  |  |  | |
| Baccalaureate or Above | Ref. |  | Ref. |  | Ref. |  | Ref. |  | Ref. |  | - |  | |
| Household Income | 0.78 | 0.73-0.83 | 0.96 | 0.78-1.18 | 0.63 | 0.59-0.67 | 0.84 | 0.78-0.90 | 0.89 | 0.76-1.05 |  |  | |
| <$30,000 |  |  |  |  |  |  |  |  |  |  |  |  | |
| $30,000-<$50,000 |  |  |  |  |  |  |  |  |  |  |  |  | |
| $50,000-<$75,000 | Ref. |  | Ref. |  | Ref. |  | Ref |  | Ref. |  | Ref. |  | |
| $75,000-<$100,000 | 0.75 | 0.69-0.82 | 1.06 | 0.84-1.34 | 80.00 | 0.73-0.88 | 0.87 | 0.79-0.96 | 0.68 | 0.55-0.84 | 0.82 | 0.54-1.25 | |
| ≥$100,000 | 0.64 | 0.59-0.70 | 0.77 | 0.59-0.99 | 0.69 | 0.63-0.75 | 0.81 | 0.75-0.89 | 0.57 | 0.47-0.70 | 0.84 | 0.56-1.25 | |
| Region | 0.35 | 0.31-0.38 | 0.60 | 0.43-0.85 | 0.37 | 0.33-0.41 | 0.55 | 0.49-0.63 | 0.32 | 0.26-0.41 | 0.50 | 0.30-0.82 | |
| Northeast |  |  |  |  |  |  |  |  |  |  |  |  | |
| Midwest |  |  |  |  |  |  |  |  |  |  |  |  | |
| South | 2.86 | 2.54-3.22 | 1.49 | 1.09-2.05 | 2.58 | 2.27-2.93 | 1.47 | 1.27-1.70 | 4.97 | 3.82-6.41 | 3.85 | 1.82-8.15 | |
| West | 2.21 | 1.95-2.50 | 1.12 | 0.80-1.56 | 2.18 | 1.91-2.49 | 1.51 | 1.32-1.74 | 3.12 | 2.36-4.11 | 2.76 | 1.34-5.69 | |
| Population Density | 1.68 | 1.47-1.92 | 0.85 | 0.58-1.23 | 1.62 | 1.40-1.86 | 1.34 | 1.16-1.54 | 2.21 | 1.63-2.99 | 1.67 | 0.77-3.61 | |
| Rural | 1.42 | 1.22-1.65 | 0.85 | 0.55-1.32 | 1.37 | 1.17-1.61 | 1.25 | 1.07-1.46 | 1.92 | 2.35-2.72 | 2.22 | 1.01-4.87 | |
| Suburban | Ref. |  | Ref. |  | Ref. |  | Ref. |  | Ref. |  | Ref. |  | |
| Urban |  |  |  |  |  |  |  |  |  |  |  |  | |
| Abbreviations: PR: Unadjusted prevalence ratio; aPR: Adjusted prevalence ratio; CI: Confidence intervals; Ref: Reference  - : This variable was not included in the final full model due to specifications outlined in the method section (p<0.10)  *Insurance variables modeled as binary (i.e., those with the specific insurance type vs. not)  All models are adjusted for survey year | | | | | | | | | | | | | |

| Supplementary Table 4: Associated Characteristics of financial hardship among cancer survivors in the COVID Impact Survey, a nationally representative survey of US, using logistic regression modeling (n=854) (April-June 2020) | | | | | | | | | | | | | |
| --- | --- | --- | --- | --- | --- | --- | --- | --- | --- | --- | --- | --- | --- |
|  |  |  |  |  |  |  |  |  |  |  |  |  |  |
|  | Overall | | | | 18-59 years | | | | 60+ years | | | |  |
|  | OR | 95% CI | aOR | 95% CI | OR | 95% CI | aOR | 95% CI | OR | 95% CI | aOR | 95% CI | |
| Age |  |  |  |  | - |  |  |  |  |  |  |  | |
| 18-29 | 1.95 | 0.71-5.34 | 1.28 | 0.38-4.31 |  |  |  |  |  |  |  |  | |
| 30-44 | 5.94 | 2.98-11.88 | 5.51 | 2.49-12.17 |  |  |  |  |  |  |  |  | |
| 45-49 | 2.19 | 1.41-3.99 | 2.64 | 1.51-4.62 |  |  |  |  |  |  |  |  | |
| 60+ | Ref. |  | Ref. |  |  |  |  |  |  |  |  |  | |
| Sex |  |  |  |  |  |  |  |  |  |  |  |  | |
| Male | Ref. |  | Ref. |  | Ref. |  | - |  | Ref. |  | Ref. |  | |
| Female | 1.94 | 1.34-2.79 | 1.50 | 0.92-2.45 | 1.47 | 0.79-2.69 |  |  | 2.05 | 1.29-3.25 | 1.95 | 1.09-3.49 | |
| Marital Status |  |  |  |  |  |  |  |  |  |  |  |  | |
| Married/Living  with Partner | Ref. |  | Ref. |  | Ref. |  | Ref. |  | Ref. |  | Ref. |  | |
| Widowed/Divorced  /Separated | 1.89 | 1.58-2.06 | 1.47 | 0.84-2.59 | 2.07 | 0.98-4.37 | 2.02 | 0.75-5.49 | 1.83 | 1.11-3.01 | 1.31 | 0.65-2.64 | |
| Never Married | 2.62 | 1.80-2.37 | 1.19 | 0.54-2.65 | 6.73 | 2.83-15.9 | 4.84 | 1.37-17.2 | 0.72 | 0.32-1.58 | 0.56 | 0.15-2.08 | |
| Race/Ethnicity |  |  |  |  |  |  |  |  |  |  |  |  | |
| White, NH | Ref. |  | Ref. |  | Ref. |  |  |  | Ref. |  | Ref. |  | |
| Black, NH | 7.34 | 3.47-15.55 | 3.45 | 1.28-9.28 | 3.97 | 0.99-15.8 | - |  | 10.78 | 4.35-26.7 | 5.96 | 1.75-18.54 | |
| Hispanic | 1.24 | 0.62-2.50 | 0.61 | 0.29-1.29 | 1.11 | 0.44-2.83 |  |  | 0.58 | 0.24-1.38 | 0.35 | 0.11-1.10 | |
| Asian, NH | 4.58 | 1.06-19.84 | 4.43 | 0.17-118.7 | 1.28 | 0.27-6.06 |  |  | - |  | - |  | |
| Other, NH | 1.43 | 0.69-4.48 | 1.12 | 0.53-2.35 | 0.35 | 0.05-2.57 |  |  | 2.31 | 1.01-5.27 | 1.29 | 0.48-3.51 | |
| Insurance Type* |  |  |  |  |  |  |  |  |  |  |  |  | |
| Purchased Plan | 0.56 | 0.35-0.87 |  |  | 0.93 | 0.35-2.47 | - |  | 0.62 | 0.37-1.06 | - |  | |
| Employer-Sponsored | 0.65 | 0.45-0.94 | 1.75 |  | 0.23 | 0.12-0.44 | 1.38 | 0.53-3.56 | 0.68 | 0.42-1.11 | - |  | |
| TRICARE | 0.34 | 0.15-0.79 | 0.43 | 0.10-1.92 | 0.40 | 0.11-1.56 | - |  | 0.48 | 0.18-1.25 | - |  | |
| Medicaid | 4.68 | 3.09-7.09 | 3.18 | 1.66-6.10 | 14.1 | 5.83-34.1 | 3.69 | 0.90-15.1 | 3.42 | 2.04-5.74 | 1.81 | 0.95-3.42 | |
| Medicare | 0.82 | 0.57-1.19 | - |  | 5.35 | 2.02-14.2 | 3.20 | 0.98-10.4 | 1.21 | 0.70-2.11 | - |  | |
| VA | 0.54 | 0.28-1.06 | - |  | 0.24 | 0.06-1.01 | - |  | 0.85 | 0.41-1.74 | - |  | |
| No insurance | 3.55 | 1.05-11.98 | 2.83 | 0.62-12.77 | 4.27 | 0.87-20.8 | - |  | 3.09 | 0.58-16.39 | - |  | |
| Any Comorbid Conditions | 2.03 | 1.27-3.26 | 1.36 | 0.77-2.42 | 1.79 | 0.83-3.87 | - |  | 2.31 | 1.25-4.27 | 1.45 | 0.61-3.47 | |
| Employment Status |  |  |  |  |  |  |  |  |  |  |  |  | |
| Not Employed | Ref. |  | - |  | Ref. |  | Ref. |  | Ref. |  | - |  | |
| Employed/Self-Employed | 0.89 | 0.60-1.30 |  |  | 0.27 | 0.14-0.51 | 0.72 | 0.27-1.92 | 0.76 | 0.44-1.29 |  |  | |
| Education |  |  |  |  |  |  |  |  |  |  |  |  | |
| No HS Diploma | Ref. |  | Ref. |  | Ref. |  | Ref. |  | Ref. |  | Ref. |  | |
| HS Graduate | 0.27 | 0.11-0.67 | 0.43 | 0.14-1.33 | 0.89 | 0.15-5.25 | 1.48 | 0.17-13.1 | 0.22 | 0.07-0.67 | 0.37 | 0.07-1.82 | |
| Some College | 0.12 | 0.05-0.29 | 0.17 | 0.06-0.50 | 0.07 | 0.01-0.38 | 0.13 | 0.02-0.85 | 0.14 | 0.05-0.43 | 0.35 | 0.07-1.61 | |
| Baccalaureate or Above | 0.06 | 0.03-0.16 | 0.08 | 0.03-0.29 | 0.03 | 0.01-0.18 | 0.09 | 0.01-0.74 | 0.08 | 0.03-0.24 | 0.12 | 0.02-0.65 | |
| Household Income |  |  |  |  |  |  |  |  |  |  |  |  | |
| <$30,000 | 6.57 | 3.62-11.90 | 2.75 | 1.24-6.09 | 13.9 | 5.15-37.8 | 2.18 | 0.54-8.68 | 14.1 | 6.68-32.3 | 4.09 | 1.41-11.91 | |
| $30,000-<$50,000 | 2.15 | 1.19-3.88 | 1.39 | 0.67-2.89 | 2.35 | 0.99-5.63 | 0.65 | 0.18-2.38 | 5.09 | 2.28-11.34 | 2.61 | 0.94-7.28 | |
| $50,000-<$75,000 | 1.20 | 0.64-2.26 | 1.02 | 0.52-2.03 | 1.55 | 0.62-3.84 | 0.77 | 0.26-2.28 | 2.74 | 1.12-6.72 | 1.60 | 0.56-4.59 | |
| $75,000-<$100,000 | 0.95 | 0.48-1.90 | 1.23 | 0.55-2.77 | 0.98 | 0.36-2.69 | 0.82 | 0.26-2.57 | 2.31 | 0.86-6.19 | 2.23 | 0.74-6.66 | |
| ≥$100,000 | Ref. |  | Ref. |  | Ref. |  | Ref. |  | Ref. |  | Ref. |  | |
| Region |  |  |  |  |  |  |  |  |  |  |  |  | |
| Northeast | Ref. |  | - |  | Ref. |  | - |  | Ref. |  | - |  | |
| Midwest | 0.88 | 0.47-1.62 |  |  | 0.81 | 0.30-2.24 |  |  | 0.99 | 0.47-2.10 |  |  | |
| South | 1.06 | 0.59-1.92 |  |  | 0.89 | 0.33-2.45 |  |  | 1.53 | 0.75-3.10 |  |  | |
| West | 0.85 | 0.47-1.55 |  |  | 0.75 | 0.28-2.00 |  |  | 0.87 | 0.41-1.82 |  |  | |
| Population Density |  |  |  |  |  |  |  |  |  |  |  |  | |
| Rural | 1.67 | 0.93-3.03 | 0.76 | 0.36-1.57 | 1.50 | 0.50-4.48 | - |  | 2.26 | 1.13-4.54 | 1.22 | 0.54-2.75 | |
| Suburban | 0.58 | 0.37-0.91 | 0.46 | 0.23-0.92 | 0.67 | 0.32-1.40 |  |  | 0.47 | 0.25-0.87 | 0.67 | 0.27-1.66 | |
| Urban | Ref. |  | Ref. |  | Ref. |  |  |  | Ref. |  | Ref. |  | |
| Abbreviations: OR: Unadjusted odds ratio; aOR: Adjusted odds ratio; CI: Confidence intervals; Ref: Reference  - : This variable was not included in the final full model due to specifications outlined in the method section (p<0.10)  *Insurance variables modeled as binary (i.e., those with the specific insurance type vs. not)  All models are adjusted for survey year | | | | | | | | | | | | | |

| Supplementary Table 5: Associations of financial hardship with mental health symptoms experienced at least one day in the past week among cancer survivors by age groups among COVID Impact Survey cancer survivors, a nationally representative survey of the US, using logistic regression modeling (April-June 2020) | | | | | | | | | |
| --- | --- | --- | --- | --- | --- | --- | --- | --- | --- |
| All Cancer Patients (n=854) |  |  |  |  |  |  |  |  |  |
|  | Overall | | | 18-59 years | | | 60+ years | | |
|  | aOR | 95% CI | | aOR | 95% CI | | aOR | 95% CI | |
| Felt Nervous, Anxious, On edge | 2.56 | 1.60 | 4.09 | 2.92 | 1.38 | 6.13 | 2.36 | 1.24 | 4.47 |
| Felt Depressed | 2.34 | 1.39 | 3.57 | 6.24 | 2.63 | 14.78 | 1.34 | 0.73 | 2.47 |
| Felt Lonely | 2.39 | 1.54 | 3.72 | 2.16 | 1.04 | 4.49 | 2.38 | 1.38 | 4.08 |
| Felt Hopeless about the Future | 1.67 | 1.10 | 2.55 | 1.36 | 0.65 | 2.85 | 1.19 | 1.15 | 3.28 |
| Cancer Patients without a self-reported diagnosed Mental Health condition (n=737) |  |  |  |  |  |  |  |  |  |
|  | Overall | | | 18-59 years | | | 60+ years | | |
|  | aOR | 95% CI | | aOR | 95% CI | | aOR | 95% CI | |
| Felt Nervous, Anxious, On edge | 1.98 | 1.17 | 3.34 | 2.21 | 0.91 | 5.38 | 1.82 | 0.90 | 3.69 |
| Felt Depressed | 1.82 | 1.08 | 3.05 | 7.82 | 2.32 | 26.37 | 1.00 | 0.51 | 1.98 |
| Felt Lonely | 2.32 | 1.43 | 3.76 | 1.57 | 0.69 | 3.56 | 2.39 | 1.32 | 4.33 |
| Felt Hopeless about the Future | 1.42 | 0.88 | 2.29 | 1.30 | 0.55 | 3.09 | 1.58 | 0.88 | 2.84 |

Models were adjusted for: age (when appropriate), survey week, sex, race/ethnicity, annual household income, education, insurance status, employment status, and area of residence (urban/rural).
